# A Case about the Upgrade of Manufacturing Equipment for Insertion into an Industry 4.0 Environment

**DOI:** 10.3390/s19153304

**Published:** 2019-07-27

**Authors:** Marcelo A. García-Garza, Horacio Ahuett-Garza, Maria G. Lopez, Pedro Orta-Castañón, Thomas R. Kurfess, Pedro D. Urbina Coronado, David Güemes-Castorena, Salvador G. Villa, Sergio Salinas

**Affiliations:** 1 Tecnologico de Monterrey, Escuela de Ingeniería y Ciencias, Ave. Eugenio Garza Sada 2501, Monterrey 64849, Mexico; 2 George W. Woodruff School of Mechanical Engineering, Georgia Institute of Technology, 771 Ferst Drive, NW, Love Bldg. Room 101, Atlanta, GA 30332-0405, USA; 3 Sisamex, S.A. de C.V. Carretera Monterrey–Colombia Km. 6, Gral. Escobedo 66050, Mexico

**Keywords:** industry 4.0, smart manufacturing, decision making, gear testing, noise analysis

## Abstract

Industry 4.0 is a synonym for the confluence of technologies that allows the integration of information technology, data science, and automated equipment, to produce smart industrial systems. The process of inserting new technologies into current conventional environments involves a wide range of disciplines and approaches. This article presents the process that was followed to identify and upgrade one station in an industrial workshop to make it compatible with the more extensive system as it evolves into the Industry 4.0 environment. An information processing kit was developed to upgrade the equipment from an automated machine to an Industry 4.0 station. The kit includes a structure to support the sensor and the data processing unit; this unit consisted of a minicomputer that records the data, graded the performance of the components, and sent the data to the cloud for storage, reporting, and further analysis. The information processing kit allowed the monitoring of the inspection system and improved the quality and speed of the inspection process.

## 1. Introduction

The term Industry 4.0 was coined to represent the latest stage in the evolution of industrial development, which has gone from the introduction of powered machines (1.0), through mass production (2.0), to computer-based automation (3.0) [1]. The new industrial facility adds the capacity to generate data and interact with the digital environment to extract and analyze data with the purpose of speeding up, or even automate, the decision-making process [2].

Automation is the main characteristic of Industry 3.0 facilities. The insertion of technologies associated with the Internet of Things, such as cloud computing, smart devices, communication tools and protocols, and digitalization of assets and products allows for the transition into Industry 4.0 [3] which is closely linked to the Knowledge Management 4.0 paradigm [4].

The task of configuring a system for the new industrial ecosystems is complex and full of risks given the investments that accompany the new systems, as well as the lack of understanding of what new capabilities are needed at any stage of the evolution. One example of this is the management of Big Data in the manufacturing shop floor [5]. In particular, the design of new facilities presents challenges, in terms of the selection of alternatives that make the best use of new technologies [6]. Companies that provide the infrastructure to facilitate the transmission, storage, and analysis of data, as well as those that produce automated, sensorized, industrial equipment, offer guidance to potential users [7]. This experience is highly valuable when designing industrial systems [8] from scratch.

In most industrial sectors, older facilities have to coexist with the new factories. That is, environments tailored in the Industry 3.0 pattern, or even older, have to interact with the new systems [9]. Countries can have a very important manufacturing industry, and still be behind state of the art sophistication in equipment [10]. This is particularly true for the metal making industry, where the life span of equipment may last decades. In these cases, it is not rare to find automated equipment that has no computer control, along with equipment that includes the latest in technological development [11]. Another very important aspect associated to this situation is the need to communicate using standards like OPC-UA [12]. Older equipment may need to be upgraded to work in the new conditions, and factories as a whole evolve into a more modern state. Engineers in charge of coordinating this evolution face themselves with the fundamental question of what the needs are and how new technologies will be adapted to allow coordination between the different technologies, in such a way that the best practices can be absorbed to remain competitive. The evolution can even point toward the change in business model, for example towards an Industrial Product Service Systems (IPSS) [13].

This article presents a case about the upgrade of manufacturing equipment for insertion in an Industry 4.0 environment, where a kit was developed to eliminate the need for a human operator who tested and validated a product’s compliance against a set of performance specifications. The case’s starting point was the analysis of the product’s fabrication system. The result of the analysis was the identification of an intermediate inspection process that was not performing with the desired reliability. Furthermore, a more detailed analysis showed that there was an opportunity to improve the quality of the information that this process provided.

During this process, different disciplines interacted to design the solution: Industrial engineering, mechanical and mechatronics engineering, information technologies, and data science. The proposed solution consisted of an information-oriented upgrade, without significant effects on the structure of the equipment. This measure contrasted with the option that is typically offered in industry: A retrofit, which focuses on the automatization of the machine and which was the characteristic action taken to modify equipment designed with Industry 2.0 principles to allow it to work in Industry 3.0 environments.

Experiences from this case provide insights into the approach that a manufacturing company can use to effectively transition towards Industry 4.0, about the choice of technological applications that can be used to interact with a Smart Factory, and about how the proposed solutions can be prioritized and scheduled.

The organization of this article is the following. Section 2 describes the methodology followed to identify the station to be upgraded, as well as the proposed architecture and design specifications for the upgrade kit. Section 3 discusses in detail the design and development of the upgrade kit. Section 4 discusses the lessons learned during the design and implementation process. Finally, Section 5 presents conclusions and future work.

## 2. Analysis of the Current System and Specification of the Solution’s Architecture 

**Figure 1:**
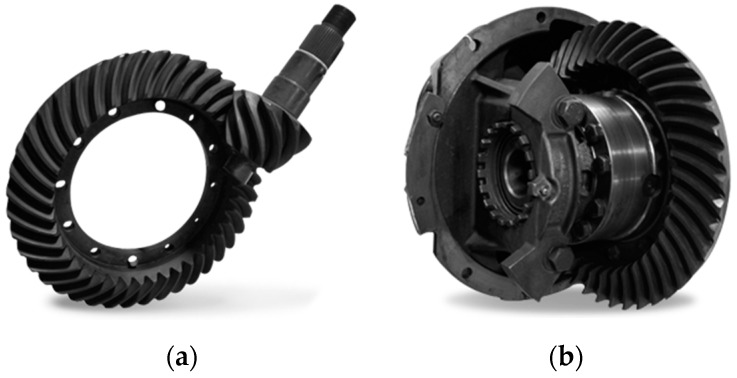
(**a**) Pinion and ring gears, (**b**) carrier differential.

This case study was developed in one of the production facilities of an automotive component’s manufacturer based in Monterrey, Mexico. The company specializes in the manufacturing of the main axle and powertrain components, such as drivelines, housings, shafts, yokes, brake components, and differentials. Each production plant has its particularities and complexity in terms of manufacturing processes, capacities, capabilities, internal and external clients and suppliers, materials, machinery, and control parameters. The study case was identified as part of the quality improvement process of the gear manufacturing plant, which produces differentials (Figure 1). A high level of complexity characterizes the production processes in this plant in terms of quality, and production planning and execution, which is inherent to the product itself.

**Figure 2:**
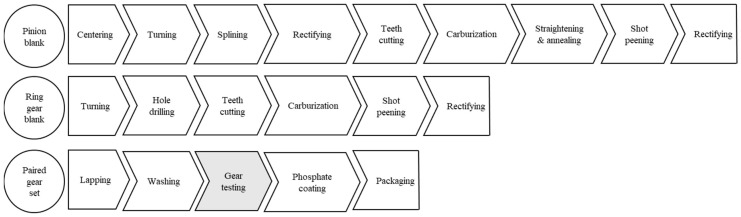
Gear set manufacturing processes.

The carrier differential’s most important gears are the pinion (driver) and ring (driven) gears. These components are responsible for transferring power from the driveline to the axle. Because of their importance in the differential’s performance, proper process monitoring is vital for their manufacturing, mainly because these gears undergo separate manufacturing processes before they get paired as a gear set. As shown in Figure 2, pinion and ring gears take different paths on the manufacturing floor. As would be expected, they have different cycle times, which altogether means that they have different lead times. This type of process is prone to imbalances and waste production, particularly under a push system.

For the above reasons, the company decided to organize a series of workshops to train their production engineers in the Toyota Production System (TPS), over six months. The goal was to develop a more organized, robust and waste-free production line, and switch their push system to a pull system, with a final goal of connecting the identified opportunities to the whole production system.

### 2.1. TPS Deployment

TPS’s main objectives are to improve quality while reducing costs and lead times. To do this, Toyota has developed a set of problem-solving and analysis tools which help identify and then execute Kaizen to solve opportunity areas [14,15,16]. The following tools were used in the analysis and evaluation process:

- Value Stream Mapping, which helped identify the current and ideal production systems.
- Standardized work which helped measure, identify and redesign operations at bottlenecks.
- Single-Minute Exchange of Die (SMED) which helped decrease changeovers and maintenance times.

Other tools, like 5s and Poka-Yoke, were reviewed as well. However, it was the A3 problem-solving methodology that helped identify an area of opportunity that led to the development of this Industry 4.0 case.

The following seven steps present the A3 process and the findings in each case:

Step 1. Problem Description

There has been a significant increase in cycle times in the lapping section for a few gear sets during a specific period (Gap). These gear sets are taking an average of about 3.5 times the normal cycle time. When gear sets go through the tester machine, which is designed to help visualize the contact between the teeth of the mating gears, they are sent back for extra lapping when they are considered as too noisy by the operator. There is a visual standard for the contact test, but there was no standard for the noise test. The gear testing machine was originally built in the 60 s. It consists of two mount heads (one for each bevel gear) whose position can be adjusted to allow for proper gear meshing. The machine is calibrated for a particular family of gear pairs (arbor shaft heights and distances), and small manual adjustments may be made during testing of a batch of gear pairs. The machine does not have computer control, and from this perspective, it can be considered pre-Industry 3.0.

Step 2. Analyze the problem

For the analysis of the problem, inspection records were consulted to identify which gear sets and how many sets presented this problem. It was found that specific gear sets with a particular cutting technology had a poor surface finish on the gear teeth. Because of this, the point of occurrence was set to study the teeth cutting operation.

Step 3. Define objectives

The objective of this project was to reduce by 30% the high cycle times on gear sets that use the current gear cutting technology.

Step 4. The 5-Whys exemplified

• Why are cycle times taking too long?

Because the machine operator is increasing the number of passes on the lapping machine.

• Why?

Because the operator observes a defective surface finish on gear teeth.

• Why?

Because surface finish quality criteria are different in lapping and tooth cutting.

• Why?

Because tooth cutting inspection is done by attributes only.

• Why?

Because there is no roughness tester to test for surface finishing.

Step 5. Containment

A set of countermeasures for problem containment were established. The criteria to choose the countermeasures was based on the expertise of the engineers and technicians in charge of the area. The cost, time and effectiveness were evaluated for each countermeasure. They are listed in Table 1.

Step 6. Action Plan

The above countermeasures were assigned to specific people depending on the specialty of each countermeasure (manufacturing, industrial engineering) as well as a specific due date. A follow-up meeting was scheduled two weeks later, to monitor each of the activities.

Step 7. Follow up

An exploratory test was conducted, and Table 2 shows the results of this test.

As a follow up on the previous activities, there were a few countermeasures that were discarded for not addressing the root cause directly. At the same time, changing cutting technology developed into a separate project, due to the project time, cost and engineering complexity. Similarly, the installation of sound meters and the definition of noise criteria developed into a specialized project as well, since experimentation showed no significant difference in noise meters for noisy gear sets.

State-of-the-art noise measuring systems and gear fault detection techniques were researched and analyzed. Gear fault diagnosis has been studied using vibrations [17] and current [18]. Other works combine acoustic and vibration measurements for gear fault diagnosis [19]. Li et al. propose the technique of periodic potential underdamped stochastic resonance to provide gear fault diagnosis from acoustic measurements in environments of high environmental noise [20]. Techniques of unsupervised machine learning have been applied to the detection of gear box bearing faults in environments of heavy background noises [21]. Given that most of the state-of-the-art measuring systems in industry are embedded in machines, solving this issue was a costly alternative; for that reason, the development of a fault-detecting and noise-measuring system resulted in a better alternative. The development of this system is explained in the next sections.

### 2.2. Specification of Information Processing Kit Architecture for Industry 4.0

From the previous analysis, the need for an information processing kit was established. The specifications for this kit were the following, considering some recommendations [22,23,24,25]:

- Eliminate subjective judgment of noisy gear sets. Before the project implementation, judgment of noisy gears was based on the operator’s perception, giving the possibility of false positives and negatives. This system lacked robustness mainly because of the inherent variation in operator’s hearing capabilities and experience. By eliminating the operator decision factor, a higher value of repeatability and reproducibility (R&R) was expected. As will be discussed, in the proposed method the noise from a test is analyzed and characterized by a series of parameters. These parameters can then be used to establish the quality of the set.
- Data storage. An essential factor in Industry 4.0 is the ability to store data for further analysis [1]. In this case the proposed control system allows for local processing of the information for quality assessment of the gears. At the same time, the system allows data collected from the process to be sent on to the Cloud and stored for further analysis. The goal is that production and quality can be monitored over longer periods of time, with the aim of setting a course for continuous quality improvement.
- Reports and quality certification sheets. A report or a quality certification sheet can be done automatically by recording the results of each gear set test into a predefined report format. An example of a prototype report sheet is shown in Appendix A.
- Productivity Analysis. The proposed design can be further developed and designed for productivity analysis capability. Calculating the overall equipment effectiveness for the operation is possible, when information about the availability of the machine, the time between tests and results of the tests is available.

## 3. Design and Development of the Upgrade Kit

This section describes the hardware and software design of the upgrade kit, as well as the implementation of the kit in the testing machine. The main components that comprise the kit are the:

- Mechanical structure: A structure manufactured with additive manufacturing technique.
- Minicomputer or wireless card: Electronic card to perform the data acquisition, perform analysis and connect to the internet. In this case, the commercial card *BeagleBone* was used for implementation.
- Microphone: Sensor used to acquire sound from the mating gears. It uses an interface to connect with the wireless card.

### 3.1. Design of Mechanical Structure

A structure was designed to support and attach the noise analysis system to the machine based on these three premises:

- The equipment must fit without interfering with the conventional operation.
- The structure should allow for sensor position adjustments.
- The structure most protect sensitive equipment from the environment.

**Figure 3:**
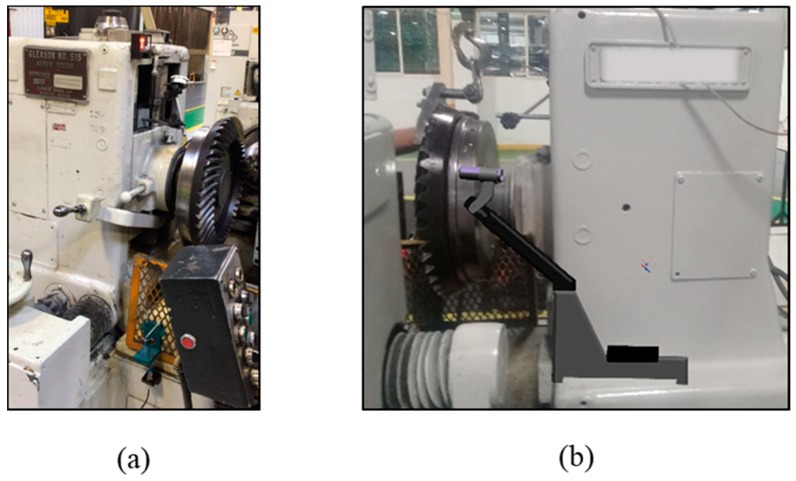
Gear testing machine. (**a**) Gear mount areas seen for the position of the operator. (**b**) Proposed location for test system with Conceptual CAD model of support.

Figure 3 shows the machine and the proposed location for the noise analysis system. There is no enclosure and therefore the test is conducted in the ambient conditions of the plant, i.e., in the presence of varying levels of noise. The design of the structure was intended to allow three degrees of freedom for placing and orienting the microphone. It was determined that the best position from which to access the test area was behind the gear set, opposite from the operator’s position. The non-moving part of the machine was selected for placement of the support structure because it presents the lowest risk for collision.

The majority of components of the structure were manufactured using a variation of the fused deposition modeling, a process that is suitable for the production of small lots or plastic parts, and that has no need for post-processing [26]. The parts were designed in NX 10.0 and fabricated with a Mark two composite 3D printer, that has a precision of +/−125 micrometers and can deposit layers of 100–200 micrometers in thickness, depending on the material. Onyx (a particle reinforced composite material) was used as the matrix, and continuous carbon fibers were deposited as reinforcement. Parts produced with this process have strengths that compete with that of metals such as aluminum [27].

**Figure 4:**
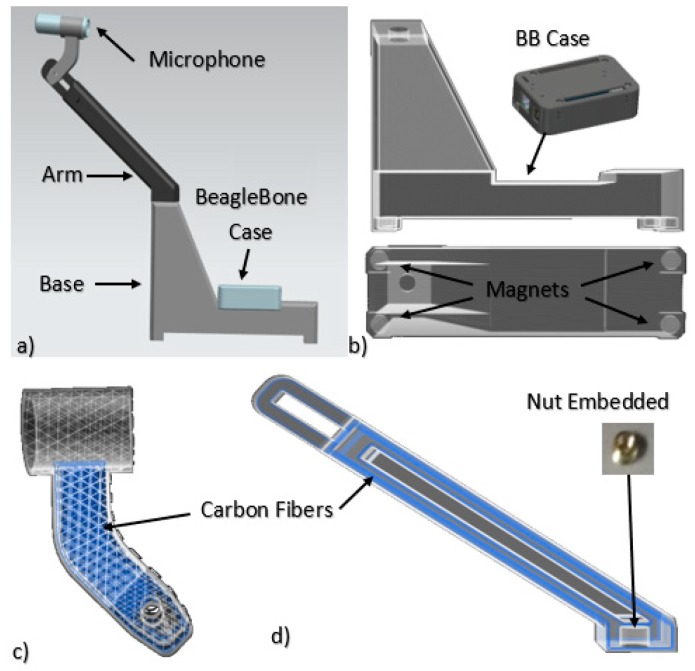
(**a**) Isometric view 3D printed assembly; (**b**) base lateral and bottom views; (**c**) pivot; and (**d**) arm.

Figure 4 shows the isometric view of the 3D printed assembly built for microphone and *BeagleBone* support. The assembly is composed of three structural parts: Arm, pivot and base and a case for the *BeagleBone*. This design used many of the advantages of additive manufacturing such as being a rapid prototype, low cost for a one-of-a-kind component and geometry design freedom [3,28]. Figure 4d shows how the arm part was reinforced with carbon fibers that increase the tensile strength of the part; also the connection nut was embedded during the 3D printing process. Similarly, the pivot component used an isotropic arrangement of carbon fibers (Figure 4c). Finally, for a secure and robust mounting design, magnets and weights were introduced inside the base part, Figure 4b, during the printing process.

### 3.2. Development of the Test for Gear Set Noise Analysis

In addition to the specification discussed in Section 2.2, other considerations were made to guarantee the functionality of the kit:

- The test set up must fit in the available space and
- Sensors and controllers must be portable

#### 3.2.1. Sensor and Equipment Selection

In the test, a highly experienced operator can detect faults in a gear set based on the noise. A microphone was the natural choice to be the primary sensor for the new test set up. A unidirectional microphone with a cardioid polar pattern, with a sensitivity of −38 dB and a 20 Hz to 20 kHz effective passband, was selected to monitor the sound produced by the contact between teeth gears in the test machine. The reasons for this selection were:

- This type of microphone is suitable for measuring localized sources without being severely affected by ambient noise.
- The cardioid polar pattern perceives frontal sound correctly, has lower sensitivity in lateral sounds and the rear sounds are rejected almost completely.
- This condenser microphone has a maximum sound pressure level of 150 dB.

**Figure 5:**
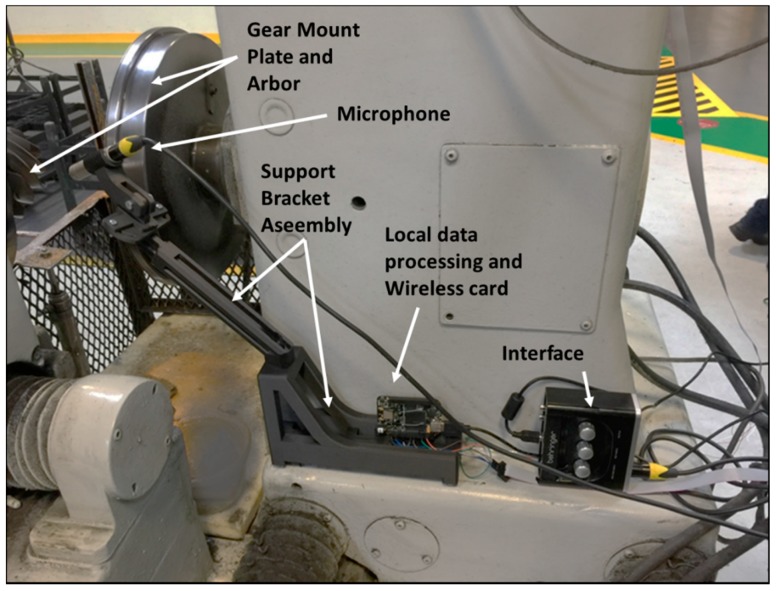
Equipment for the audio signal monitoring system: Support bracket assembly unidirectional microphone, interface, and BeagleBone wireless card.

During initial tests, the sensor was held by a magnetic base in the back of the machine, in an area that does not interfere in the process nor the access of the pieces and allows locating the microphone at a very close distance (~1.5 cm) and perpendicular to the area of contact between the teeth (see Figure 5). This position allows obtaining the predominant signal of the teeth gear contact while reducing the phenomenon of masking that occurs in very noisy environments. The microphone connects to a professional audio interface with 48 kHz resolution, which provides a phantom power (+48 V DC) required by the microphone. The output of the audio interface is connected via USB cable to the *BeagleBone*, which in turn, has a wireless internet connection.

**Figure 6:**
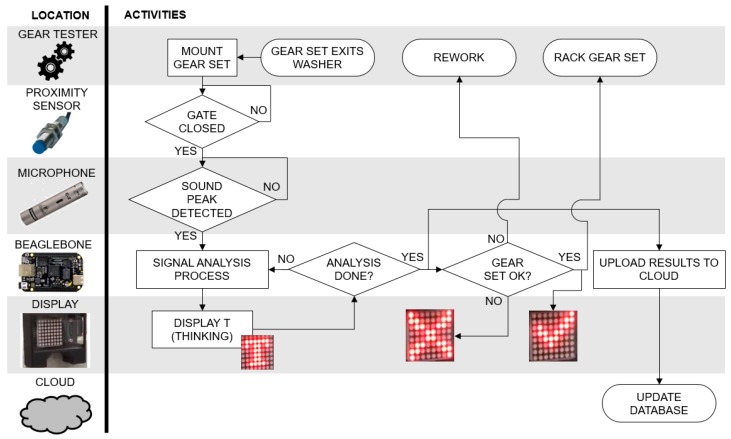
General view of the gear noise test system’s architecture and operation. The gears go through a washing process prior to the noise test, which starts when the gears are mounted.

Figure 6 presents the general system’s architecture and operation. The functions and activities implemented to evaluate the gear set’s performance are shown in the flowchart and are correlated with the hardware components that perform each function.

#### 3.2.2. Data Collection and Analysis Procedure

The need to extract useful information from big data is at the core of Industry 4.0. The goal of this work was to report how many sets of gears have faults from a test that does not affect production times. Different techniques exist for fault diagnosis based on acoustic signals. For example, Qu et al. [29] reported a comparison between acoustic emission sensors and vibrations based on features as the root mean square (RMS). Peak to peak value and Kurtosis extracted from time synchronous averaging signals of the gearbox were used in their work. They concluded that acoustic emissions have a better and more stable performance to detect small tooth damage in the low-speed range compared to vibrations using accelerometers. Zhong et al. [30] present a rolling bearing fault diagnosis based on short time Fourier transform of audio recordings.

**Figure 7:**
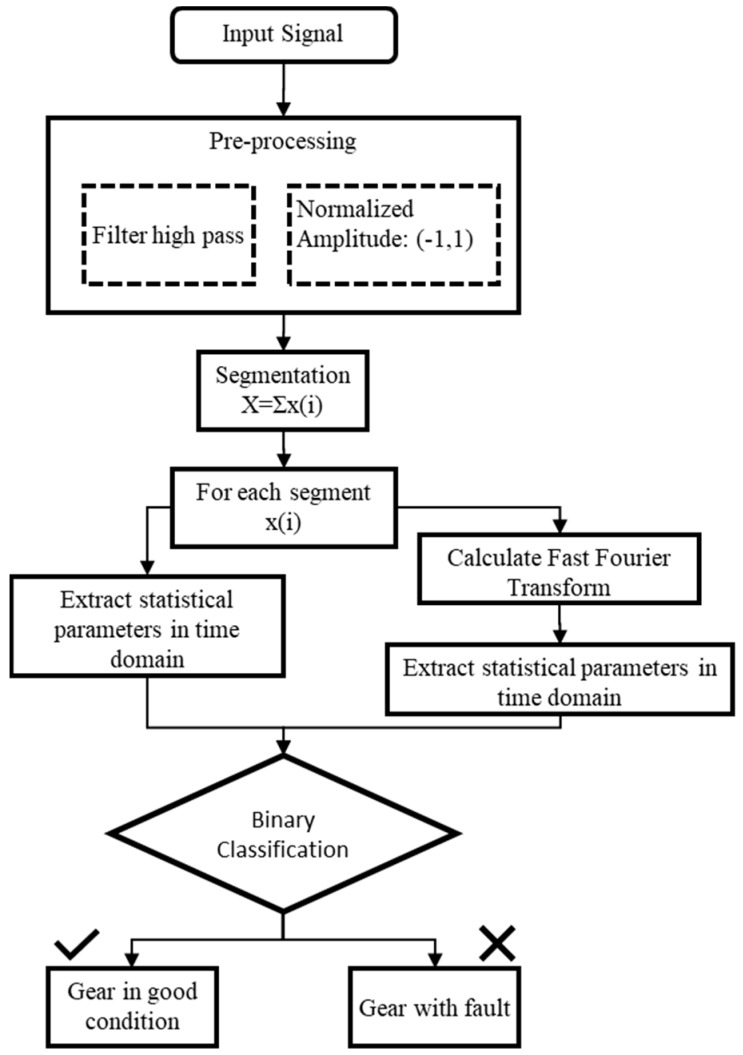
Procedure to test if a gear set is in good condition or has a fault. Data processing is shown.

In the proposed system, the audio signal is acquired for analysis. This consists of a filtering and segmentation stage for extracting the features that allow identifying if the gear set presents any faults. After the analysis, the board sends the results and other parameters useful to identify the gear set test to the cloud to make the report. Figure 7 shows the analysis procedure used in this system.

The pre-processing stage consists of filtering the signal to attenuate the low frequencies, using a high pass filter with a cutoff frequency of 100 Hz. A moving average filter was applied to reduce the noise or outlier sounds that could be captured by the microphone. Also, it was necessary to normalize the amplitude of the signals in a range of (−1, 1) to extract meaningful statistical features. The next step divides the signal into segments with the duration in the time necessary to produce all possible contacts between the teeth of the pinion and the ring gear. In this way, any anomaly that occurs in the teeth may be detected. All computations are performed locally by a program written in Python.

Feature extraction in time and frequency domain is applied for each segment of the audio signal [31,32]. Time domain statistical features can be reflecting the mechanical faults [29,33]. Standard deviation (Equation (1)) is a measure used to quantify the amount of variation of a set of data; the RMS (Equation (2)) value reflect the vibration amplitude and energy of a time domain signal—where *x*(*n*) is a signal series for *n* = 1, 2, …, *N*, and *N* is the number of data points.

(1) $$\sigma =\sqrt{\frac{\sum_{n=1}^{N}{\left(x\left(n\right)-\bar{x}\right)}^{2}}{N-1}}$$

(2) $$RMS=\sqrt{\frac{\sum_{n=1}^{N}x{\left(n\right)}^{2}}{N}}$$

Kurtosis (Equation (3)) and impulse (Equation (4)) factor can measure the impulse existing in vibration signals; the impulse factor is also a good indicator of spikiness of the sharp impulses generated by the contact of a defect in the surfaces.

(3) $$Kurtosis=\frac{\sum_{n=1}^{N}{\left(x\left(n\right)-\bar{x}\right)}^{4}}{\left(N-1\right)\sigma^{4}}$$

(4) $$Impulse=\frac{\max\left|x\left(n\right)\right|}{\frac{1}{N}\sum_{n=1}^{N}\left|x\left(n\right)\right|}$$

Also, the statistical features in the frequency domain provide useful information to detect a fault. The mean frequency feature *p*_1_ (Equation (5)) characterizes the vibration energy in the frequency domain, which represents the average of the amplitudes over all the frequencies, where *s*(*k*) is a spectrum for *k* = 1, 2, …, *K*, *K* is the number of spectrum lines and *f_k_* is the frequency value of the kth spectrum line.

(5) $$p_{1}=\frac{\sum_{k=1}^{K}s\left(k\right)}{K}$$

Feature *p*_2_ (Equation (6)) shows the position change of the main frequencies, which are dominant in the frequency spectrum.

(6) $$p_{2}=\frac{\sum_{k=1}^{K}{\left(s\left(k\right)-p_{1}\right)}^{2}}{K-1}$$

Feature *p*_3_ (Equation (7)) introduces a measure for the average frequency, while *p*_4_ (Equation (8)) describes the convergence of the spectrum power, reflecting the energy of the frequency spectrum. Feature *p*_5_ (Equation (9)) represents a ratio of the parameters *p*_4_ and *p*_3_, and is useful to differentiate between audio signals with faults and those in good condition. These statistical features in time and frequency domain were selected to achieve accurate fault diagnosis results based on audio signals.

(7) $$p_{3}=\frac{\sum_{k=1}^{K}f_{k}s\left(k\right)}{\sum_{k=1}^{K}s\left(k\right)}$$

(8) $$p_{4}=\sqrt{\frac{\sum_{k=1}^{K}{\left(f_{k}-p_{s}\right)}^{2}s\left(k\right)}{K}}$$

(9) $$p_{5}=\frac{p_{4}}{p_{3}}$$

To detect the condition of a gear set in the test, a binary classifier was used based on specific threshold values that allow identifying between a signal with a fault and a signal in good condition. The next section provides the details of the binary classifier, as well as the results of the audio signal analysis upon which the classifier was based.

#### 3.2.3. Results of the Audio Signal Analysis

Audio signals were acquired at a frequency sampling of 44,100 kHz with a duration of 30 s in a backward direction. It should be noted that audio patterns are different in amplitude and frequency spectrum for each direction of rotation (forward and backward) of the test machine. The more unambiguous results were obtained in the backward or reverse rotation direction.

**Figure 8:**
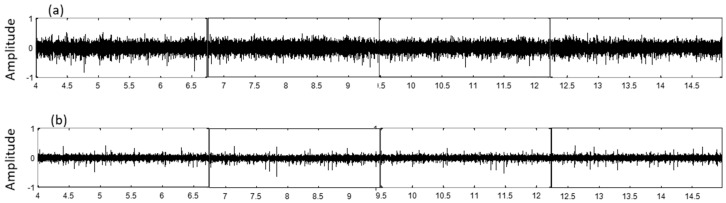
Segments of the audio signal in time domain corresponding to the gear set in (**a**) good condition and (**b**) with fault.

Figure 8 shows the comparison of the first four consecutive segments of a signal in good condition and a signal of a gear set with fault, in the time domain. The duration of each segment is 2.7 s, which is the time it takes for all the contacts between the teeth of the two gears to occur. In this case, the pinion had 10 teeth, and the ring gear had 43. The pinion rotation speed was 945 rpm, and the corresponding gear frequency was 157 Hz. The first segment started in second #4 to analyze the signal when the speed of the machine was stable. It was observed that the main difference is a decrease in the amplitude and more relevant peaks appear in the signal with failure (right side). These differences are exposed in the features extracted in the time domain, using Equations (1)–(4).

**Figure 9:**
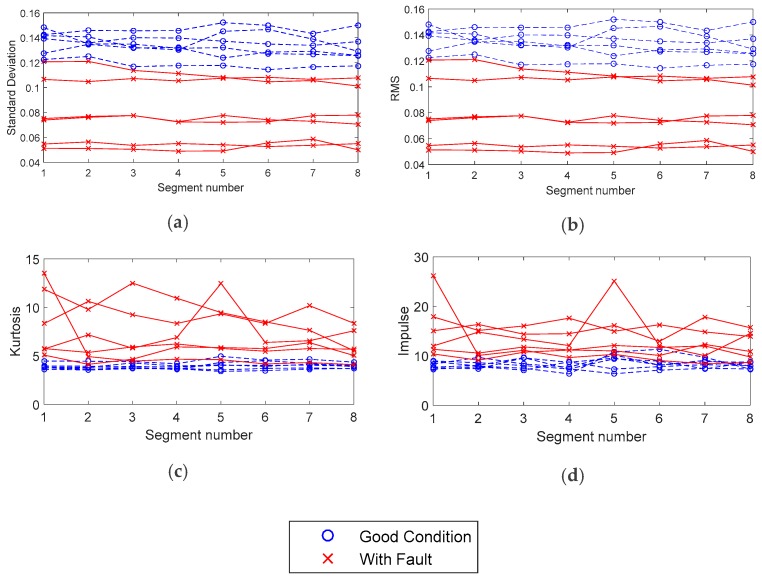
Statistics in the time domain, (**a**) standard deviation, (**b**) RMS value, (**c**) kurtosis and (**d**) impulse factor for gear sets in good condition and with fault.

Figure 9 shows the statistics extracted in the time domain. It is shown that the amplitude decreases in the audio signal from defective gears. This can be caused by the normalization process of the data, or by environmental factors such as plant noise. This behavior is different from the effect observed in the analysis of vibrations measured with accelerometers where amplitude generally increases when a gear failure occurs [33,34].

The measurements shown correspond to several tests of two gear set models, with 10–43 and 12–43 teeth of pinion-ring gears, with gear frequencies of 157 Hz and 189 Hz, respectively. In the case of the kurtosis and the impulse factor, its value increases for signals with a fault; this indicates that the signal has peaks and immediate impulses produced by imperfections in the surface of the teeth. These statistical values are used for the detection of faults in gears by wear, using vibration signals with outstanding results. For example, Fan et al. [35] reported a gear damage diagnosis and classification using support vector machines based on statistical parameters, in the time domain.

**Figure 10:**
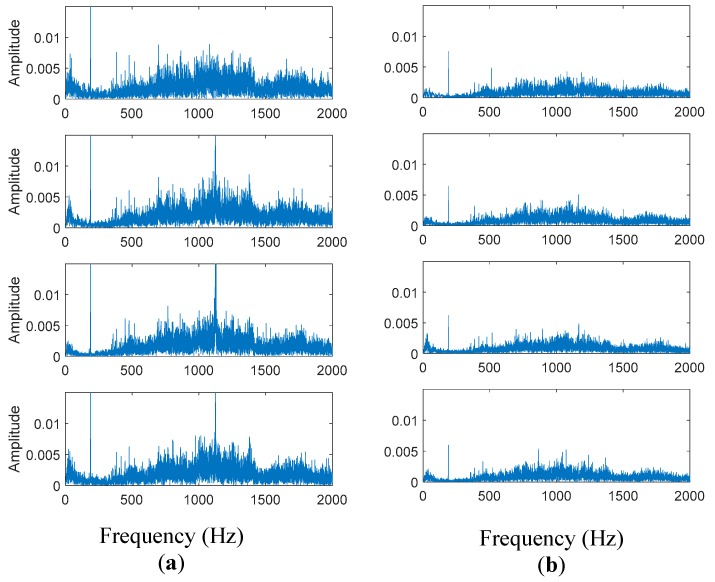
The frequency spectrum of the audio signal of a segment in time domain corresponding to the gear set in (**a**) good condition and (**b**) with fault.

Figure 10 presents the statistical values in the frequency domain. In accordance with the differences observed between the audio signals in good condition and with a fault in the time domain, it is observed that the amplitude of the frequency spectrum and energy of the gears in good condition (left side) are higher than the parameters when a fault is present in the surface of the teeth. Also, the amplitude of gear frequency at 189 Hz is higher than the other frequency components, and its amplitude is more prominent compared with a spectrum of a gear set with a fault (right side).

**Figure 11:**
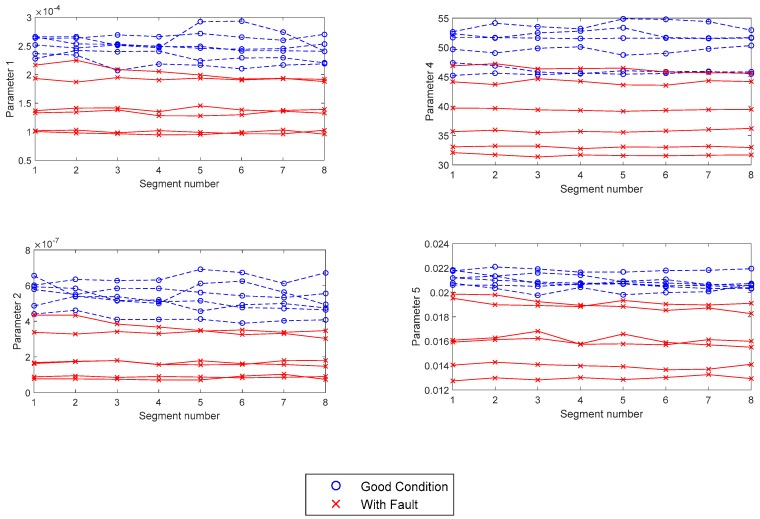
Statistical parameters 1, 2, 4, and 5 in the frequency domain to gear sets in good condition and with fault.

Considering the differences between the frequency spectrum in good condition and with fault, the statistical parameters were calculated using Equations (5)–(9), and Figure 11 shows these statistics. These features are useful to identify gear sets with faults using a threshold valid for gear sets with the same physical properties as the gears tested in this work. Similar statistical measures in time and frequency domain were used in Reference [36] from vibration signals to fault identification and classification of a gearbox.

To identify gear sets with fault, a binary classification was implemented [37,38] based on the threshold values that allow separating the statistical features extracted in time and frequency domain as shown in Table 3. The accuracy achieved in the classification for the measurements taken from two different gear models (10–43 and 12–43 teeth ratio), two conditions (good and with fault) and in different environmental noise conditions is higher than the mean accuracy achieved based on the noise perceived by the operators with great experience, as discussed in Section 2.

### 3.3. Connectivity to the Internet: From Local Data to Cloud Computing

**Figure 12:**
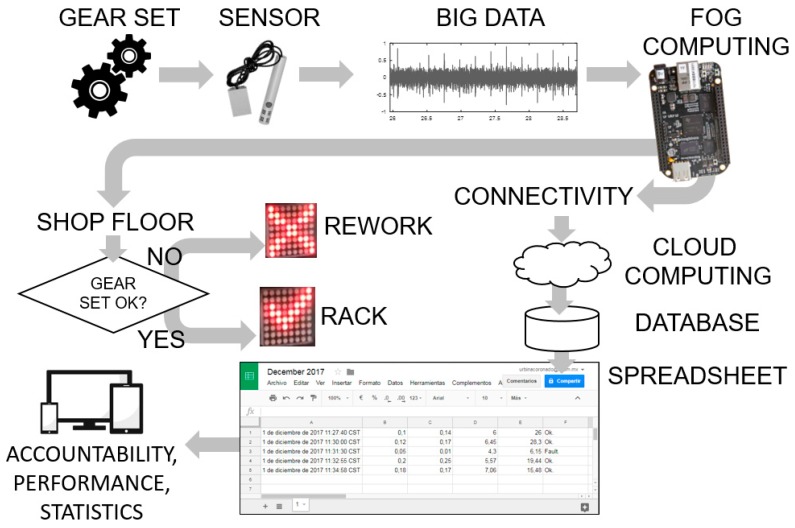
Diagram of the flow of information in the smart manufacturing gear set monitoring system. A better view of a typical report spreadsheet can be seen in the Appendix A.

Connectivity and the capability to upload data to the internet are essential parts of industry 4.0 and smart manufacturing. The operations performed in the *BeagleBone* constitute a fog-computing node. In the environment of the industrial internet of things, some of the functions of fog computing include connectivity between physical devices and network, low latency, scalable computing and real-time big data analytics [39]. Figure 12 shows a diagram with the general idea of the role of the concepts of fog computing, connectivity and cloud computing in the evolution towards Industry 4.0 for the case of this work.

The sensor capturing the sound of the gear set is producing big data. The treatment of the big data follows an approach similar to what Ji and Wang reported [7]. The fog computing node deals with the data locally produced in the sensor. The interest in the local data resides in the real-time feature extraction, the machine learning analytics, and the gear set diagnosis. The cloud computing deals with the historical data and is interested in patterns over time of the performance of the gear sets and the elaboration of reports.

The script in the *BeagleBone* makes an http post request each time a gear set is analyzed, which includes the information of each of the features extracted and the diagnosis of the gear set. The request is sent to a web app hosted in a cloud web service. In this work, the web service chosen was Google Drive, and the web app was published using Google Apps Script. The reason for choosing this platform is twofold: First, the simplicity of use (free and programmable in JavaScript-based language) and second, the company was already familiar with other products of the platform. The use of a web service (also known as big data cloud platforms) managed by a specialized third party is highly advisable for security reasons [40].

Upon receiving the information, the web app organizes and stores the data in spreadsheets. A spreadsheet is automatically created for each month, and a sheet per day is created inside the spreadsheet. The user can access the specific date and have the information of the gear sets tested and the results. One more feature enabled by this transformation is the ubiquitous access: Independently of the geographic point or the device, the information is always available. Figure 12 illustrates the flow of information in the system. In parallel operations, the decision to reprocess or accept a gear set is made at the shop floor, while the information for accountability, performance and statistics is stored and accessed in a spreadsheet.

## 4. Discussion

In regards to the data analysis process, Figure 8, Figure 9 and Figure 11 show typical parameter values extracted from the data of the gear tests. In these tests, faulty gear sets showed lower values of RMS and Standard Deviation. Furthermore, these parameters behaved fairly uniformly through all the segments. On the other hand, faulty gears showed higher values of kurtosis and impulse, which varied significantly particularly in segments 1 and 5. In general, the RMS value is a measure of the energy of the signal, which is affected by factors such as ambient noise and location of the microphone. This would explain why equipment such as sound meters are considered unreliable for these tests by operators. The processing of the data also has an effect on the RMS value, as the amplitudes are normalized to fit between (−1, 1). As explained before, the Kurtosis and impulse values on the other hand seemed to be better indicators for the presence of spikes in the signal, which accompany certain types of manufacturing errors in the gear teeth, such as pits and bumps.

A similar behavior was observed in parameters 1–5. Faulty gears reported consistently lower values. For the purposes of the quality test, what is important is that a threshold value was observable in all parameters, except for one case involving parameter 2. For this reason, the evaluation takes all parameters, statistical and those associated with frequency spectrums, into consideration.

Currently, the data processing kit has been tested in the plant. Data has been collected, analyzed and sent to the cloud. From this data, information has been extracted to monitor the test system’s performance from a computer or even mobile devices. It is important to note that the type of errors that are identified by the system are generally caused in the previous station (washing of the gears). A statistic of their occurrence can now be monitored.

From a methodological perspective, this exercise was unique in the way the integration of techniques from different disciplines were used to identify and upgrade a machine that can help the manufacturing floor productivity.

The design of the structural components proved to be flexible and robust for the application. In particular, the design has the advantage that it is easily oriented and fixed to the frame of the current machine. Furthermore, no structural modifications were needed to adapt the monitoring system to the current equipment.

One of the major challenges was to establish the correct filtering procedure for raw noise data. That is, because of the ambient conditions, the signal from the test was confused with the rest of the plant noise. Significant work was needed to obtain parameters that could discriminate the good gear set from the defective one. Initial results show that the system is capable of detecting faults in a manner that is consistent with what a trained human operator would conclude. Nevertheless, a statistical test still needs to be developed to validate the robustness of the system’s performance.

Another challenge was to determine what kind of data should be sent to the cloud for further analysis. A normal test of 30 s at 44 kHz generates a significant amount of data. Sending it all to the cloud would be too expensive in terms of bandwidth and storage capacity. In the end, the decision was made to upload only the date, hour and parameter data from the filtering process.

Finally, while some intelligence was built into the analysis, in terms of the capacity to monitor and report parameters of relevance on a timely basis or upon demand, the part tracking and correlation with the data is not yet possible, as the gear sets are not labeled individually. A next step would be to add equipment to allow marking of the gear sets when the passed the test, in such a way that the test data can be correlated with a particular product, since the company produces many different products.

## 5. Conclusions and Future Work

This article presented the procedure that was followed for the design and implementation of an information processing kit that upgrades a conventional machine and allows it to be integrated into an Industry 4.0 environment. The procedure resulted in a design that is an upgrade of the system, which is information-driven, as opposed to a retrofit, which is hardware driven. The study showed the potential to have a systemic view that incorporates lean techniques to identify information bottlenecks, i.e., processes that are not maximizing their capacity to provide data, and produced a new process, installed in plant, compatible with Industry 4.0 trends.

The upgrade kit includes a structure to support the sensor and the data processing unit; this unit is made up of a minicomputer that records the data, grades the performance of the components, and sends the data to a website for storage, reporting, and further analysis for decision-making. The exercise began with an analysis based on continuous improvement methodologies and finished with a system that was added to a particular conventional machine that allows for data from a test to be collected and uploaded to the cloud, from which decisions can be made.

The study described a structured procedure that manufacturing companies can use to effectively transition towards Industry 4.0. In particular, the process for selecting technological applications that can be used to allow an older system to perform functions that are associated with a smart factory was discussed.

Findings of this work should be of interest to manufacturing companies that are making a transition from a “brown field” to facilities that take advantage of the age of information. The experiences from this exercise can help answer typical concerns of the people responsible for running a shop. In particular, the procedure that was followed can help establish what equipment needs to be upgraded and for what purpose. While there are many approaches, this paper provided a method that can be used from an industrial, mechanical, and engineering perspective.

Future work includes the statistical validation of the test and the implementation of specific data analysis routines for each product. Similarly, the design/selection and implementation of a gear marking system for product traceability would increase the value of the information generated by the gear testing system.

## Figures and Tables

**Table 1:** Countermeasures analysis.

Countermeasure	Cost	Time	Effectiveness
Set a standard for the number of passes in lapping	Low	Medium	Low
Define noise criteria for gear testing machines	Low	Low	Medium
Install sound meter on gear testers	Medium	Medium	Medium
Implement roughness meters on cutting	High	High	High
Change cutting technology for particular models	High	High	High

**Table 2:** Effect of Lapping passes on Noise Experiment.

Gear Tester			Noise	
Gear Set	No. of Lapping Passes	Gear Contact Area	Operator’s Judgement	Sound Meter (Max. 90 dB)
1	14	OK	OK	81–86
2	9	OK	Not OK	85–87
3	7	OK	Not OK	86–88

**Table 3:** Threshold values for identifying a gear set with a fault in binary classification.

Time Features	Threshold Value	Frequency Parameters	Threshold Value
Standard deviation	≤0.12	Parameter_1	≤2 × 10^−4^
RMS	≤0.12	Parameter_2	≤4 × 10^−7^
Kurtosis	≥5	Parameter_4	≤45
Impulse	≥10	Parameter_5	≤0.02

**Figure A1:**
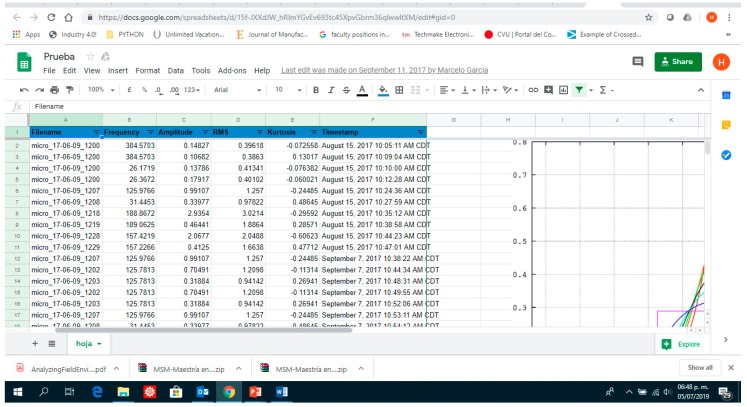
Prototype test result sheet uploaded to the cloud mentioned in Section 2.2 and Figure 12.
